# Consumption of organic food by children in Germany – Results of EsKiMo II

**DOI:** 10.25646/6399

**Published:** 2020-03-04

**Authors:** Marjolein Haftenberger, Franziska Lehmann, Clarissa Lage Barbosa, Anna-Kristin Brettschneider, Gert B. M. Mensink

**Affiliations:** 1 Formerly Robert Koch Institute, Berlin Department of Epidemiology and Health Monitoring; 2 Robert Koch Institute, Berlin Department of Epidemiology and Health Monitoring

**Keywords:** ORGANIC FOOD, FOOD CONSUMPTION, CHILDREN, ESKIMO II, KIGGS WAVE 2, HEALTH MONITORING

## Abstract

Data from the second Eating study as a KiGGS module (EsKiMo II, 2015–2017), are used to determine the contribution of food produced by organic farming (organic food) to the diet of children aged between six and eleven years (n=1,190) in Germany. Dietary intake was assessed by food records during a total of four days. Information on the proportion of organic food intake relative to daily food intake was used to differentiate between three groups: children who did not consume organic food; children whose diet contains 8.0% or less of organic food; and children whose diet comprises more than 8.0% of organic food. The 8.0% threshold represents the mean proportion of organic food eaten by children whose diet includes any amount of organic produce. In total, 63.2% of children eat organic food. The diet of 43.0% of children contains 8.0% or less of organic food, with the diet of 20.2% comprising more than 8.0% of organic food. Vegetables and fruit are among the most commonly consumed organic products. While consumption frequency of organic food does not differ by sex or age, consumption frequency increases with higher socioeconomic status. The large proportion of children (63.2%) who eat organic food suggests that health, environmental and ethical motives play a role in the food choices made by families with children.

## Introduction

Organic foods are grown using organic farming methods; the requirements that these products have to meet to be labelled as such are set out in the European regulation on organic products [1]. In 2010, a binding EU-wide labelling system was introduced for organic products (the EU organic logo). In addition, a state-regulated system and private labelling systems are also established in Germany. In some cases, the requirements governing the use of these systems are stricter than those stipulated in the EU regulation. Labelling organic foods can enable consumers to make informed food choices.

The range of organic foods available is steadily rising. Over the last 20 years, sales of organic food have multiplied throughout the world. In 2017, organic food occupied a 5.1% market share in Germany [2]. Health-related and ethical motives, such as animal welfare and protecting the environment, are among the main reasons that people buy organic food [3–6]. The German National Nutrition Survey II (NVS II, 2005–2006) found that 44.9% of adults aged between 18 and 80 occasionally or regularly bought organic produce. Compared to people without organic purchase, the dietary intake of people who buy organic foods is more closely in line with the German Nutrition Society’s dietary recommendations. People who buy organic foods also have healthier lifestyles in terms of smoking and physical activity and a lower body mass index compared with people who do not buy organic foods [5, 7].

**KiGGS Wave 2**

Second follow-up to the German Health Interview and Examination Survey for Children and Adolescents

**Data owner:** Robert Koch Institute

**Aim:** Providing reliable information on health status, health-related behaviour, living conditions, protective and risk factors, and health care among children, adolescents and young adults living in Germany, with the possibility of trend and longitudinal analyses

**Study design:** Combined cross-sectional and cohort study

**Cross-sectional study in KiGGS Wave 2**

**Age range:** 0–17 years

**Population:** Children and adolescents with permanent residence in Germany

**Sampling:** Samples from official residency registries - randomly selected children and adolescents from the 167 cities and municipalities covered by the KiGGS baseline study

**Sample size:** 15,023 participants

**KiGGS cohort study in KiGGS Wave 2**

**Age range:** 10–31 years

**Sampling:** Re-invitation of everyone who took part in the KiGGS baseline study and who was willing to participate in a follow-up

**Sample size:** 10,853 participants

**KiGGS survey waves**

- KiGGS baseline study (2003–2006), examination and interview survey
- KiGGS Wave 1 (2009–2012), interview survey
- KiGGS Wave 2 (2014–2017), examination and interview survey

More information is available at www.kiggs-studie.de/english

In the first nationwide representative nutritional study of children and adolescents, EsKiMo I, a module of the German Health Interview and Examination Survey for Children and Adolescents (KiGGS baseline study, 2003–2006), more than half (about 56%) of parents of 6- to 11-year-olds stated that they had bought organic food [8]. Although purchasing behaviour is an important determinant of consumption, the amount of organic food consumed by individuals and which household members consumed organic foods remained unclear.

Analyses on food consumption provide information about the actual contribution of organic food to the total diet of children. A higher proportion of organic food provided to their own children is an indicator that parent’s purchasing behaviour is particularly (health-)conscious. In addition to the expected health benefits, animal welfare and environmental motives also play a role when purchasing organic food. Furthermore, information on the contribution of organic food to the diet is important for future risk assessments. Organic products generally contain lower levels of pesticides [4, 9] and veterinary drug residues [10]. The use of synthetic additives in the production of organic products is also minimal [9]. In addition, there may also be differences between organically and conventionally produced foods in terms of nutrient composition and levels of other active substances such as antioxidants and polyphenols [4].

Nevertheless, there are very few population-based studies of organic food intake. The French NutriNet-Santé cohort study (2009–2011) found that about two-thirds of adult women and three-fifths of adult men consumed organic foods occasionally or frequently [11, 12]. Additionally, this study observed a negative correlation between a high intake of organic food and the incidence of metabolic syndrome [13]. However, no extensive population-based studies have been available on children’s dietary intake of organic food in Germany until now.

The second Eating study as a KiGGS module (EsKiMo II, 2015–2017), provides data on the dietary intake of organic foods among children aged between six and eleven years. In the following, the results are described in relation to sex, age and family socioeconomic status (SES).

## Indicator

EsKiMo II (2015–2017) was carried out as a module of the second follow-up survey to the German Health Interview and Examination Survey for Children and Adolescents (KiGGS Wave 2, 2014–2017). KiGGS is part of the health monitoring system at the Robert Koch Institute and includes repeated cross-sectional surveys of children and adolescents aged between 0 and 17 (KiGGS cross-sectional study) that are representative for Germany. The KiGGS baseline study (2003–2006) was conducted as an examination and interview survey, KiGGS Wave 1 (2009–2012) as a telephone-based interview survey and KiGGS Wave 2 (2014–2017) as a combined examination and interview survey. The concept and design of KiGGS Wave 2 have been described in detail elsewhere [14, 15]. Between June 2015 and September 2017, 2,644 children and adolescents aged between 6 and 17 who had participated in the cross-sectional survey of KiGGS Wave 2 also took part in EsKiMo II. Detailed information on EsKiMo II is available elsewhere [16–18]. In EsKiMo II, food consumption of children aged between six and eleven was assessed by food records [16–18]. The records included a separate column to provide information about whether foods were organic. In addition, a dietary history interview was conducted with 12- to 17-year-olds [16–18]; however, the interview did not record any data about organic food. The analyses set out below are based on data from 1,190 children aged between six and eleven (584 girls, 606 boys).

**EsKiMo II**

Second Wave of the Eating study as a KiGGS Module, 2015–2017

**Acronym: EsKiMo –** Eating study as a KiGGS Module

**Implementation:** Robert Koch Institute

**Aim:** Providing an up-to-date representative overview of the dietary habits of children and adolescents aged 6 to 17 in Germany.

**Study design:** Cross-sectional study based on a modified diet history interview and food records

**Population:** Children and adolescents with permanent residence in Germany

**Sampling:** EsKiMo II participants are randomly selected from the cross-sectional sample of KiGGS Wave 2 (registry office sample). Being invited to EsKiMo II requires participation in KiGGS Wave 2.

**Age range:** 6 to 17 years

**Sample size:** 2,644 participants

**Survey period:** June 2015 – September 2017

More information in German is available at www.rki.de/eskimo

The dietary assessment among the 6- to 11-year-olds included two food records (‘weighed records’), a three-day and a one-day record, covering a total of four days. The records were filled out by parents or guardians after a personal instruction by trained nutritionists. In addition to detailed information about each food consumed, including brand names and quantities, also information about whether the food was organic, should be recorded. During the instruction on the food records, parents were informed about the EU’s and Germany’s organic food labelling systems and their respective logos. Copies of the logos were also included in the column where the parents should state whether a food was organic.

The proportion of the children’s diets that consisted of organic food was calculated by dividing the daily intake of organic food (grams per day) by the total daily intake (grams per day). The corresponding indicator is based on the mean proportion of organic food eaten during the days covered by the food record for children who did consume organic food. The indicator was defined as follows: 1) No dietary intake of organic food; 2) Dietary intake of organic food of ≤8.0%; 3) Dietary intake of organic food >8.0%. The 8.0% threshold is the mean percentage of organic food eaten by children who do consume organic food.

The study also reported within which food groups foods of organic origin are frequently consumed. In order to do so, in EsKiMo II each food recorded during the study period was assigned to one of 29 groups and these groups were classified according to consumption frequency of organic foods. This made it possible to identify the proportion of children who ate the ten most frequently cited organic foods.

Family SES was measured through a multidimensional index based on the information parents provided on educational background, occupational status and equivalised household income. The SES index allows for a differentiation between low, medium and high status groups [19].

The results are presented as frequencies stratified by sex, age and SES of the family [19]. The calculations were performed using a weighting factor that was developed for EsKiMo II. This weighting factor corrects for deviations from the population structure with regard to regional structure (rural area/urban area), age (in years), sex, federal state (as of 31 December 2015), German citizenship (as of 31 December 2014), parental level of education (Microcensus 2013 [20]), and differences in participation in the dietary survey associated with seasonality, SES of the family and the child’s school type. This article presents the results as frequencies with 95% confidence intervals (95% CI). The precision of frequencies can be assessed using confidence intervals; wide confidence intervals indicate a greater statistical uncertainty in the results. The differences in the frequencies of the proportion of organic food consumed were analysed by sex, age and SES using a chi-square test. A statistically significant difference between groups is assumed when the corresponding p-value, once the weighting factor and the survey design have been taken into account, is smaller than 0.05.

## Results and discussion

Organically produced foods play a role in our diets already from a young age. In total, 63.2% of children between the ages of six and eleven consume organic food; about one third of these (21.0%) do so on a daily basis. On average, 8.0% of the food eaten by children who do consume organic food is produced by organic farming. 43.0% of children have a dietary intake of organic food amounting to 8.0% or less; whereas 20.2% have a dietary intake of more than 8.0% of organic food. An average intake of 8.0% (median 4.0%) among children who eat organic foods seems plausible, as organic food occupied a market share of 5.1% of total sales in 2017 [2].

The frequency distribution of the proportion of organic food consumed did not differ by sex or age group (Table 1). However, a social gradient was identified: children from families with a low SES are most often non-consumers of organic products, whereas the proportion of children whose diets consist of more than 8.0% of organic food increases significantly with rising SES (Table 1). Similar observations were made by the French NutriNet-Santé study. The French study found that the proportion of adults who regularly consume organic food increased with education, occupational status and income [12]. In addition, the German NVS II study found that adults who purchased organic food more often had a higher SES than those who did not [5, 7]. Presumably, price acts as a barrier to low-income households for the purchase of organic food; as is indicated by multivariate analyses from NVS II [7]. The observation of a larger proportion of organic food among children from families with a high SES suggests that education and income play a role in children’s consumption of organic food.

**Figure 1:**
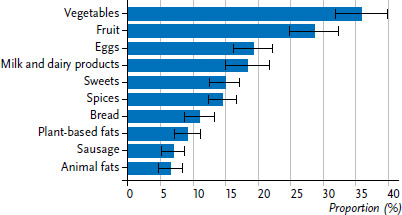
Frequency of consumption of the ten most frequently reported organic food groups (n=1,190) Source: EsKiMo II (2015–2017)

In addition to identifying the groups who eat the most organic food, EsKiMo II also demonstrates of which food groups organic foods are frequently consumed. Figure 1 shows the proportions of consumers who eat the ten most frequently consumed organic foods. Foods of plant origin, such as vegetables and fruit, are among the most commonly reported organic foods; they form part of the diets of 35.9% and 28.7% of children, respectively. It should be noted that organic meat and processed meat are rarely reported and that organic meat does not appear among the ten most frequently consumed organic foods. Results from the first EsKiMo study (2006) also show that organic meat was seldom purchased and that organic fruit and vegetables were bought more often (by parents of children aged between six and eleven) [8].

When interpreting the results, it is important to consider that the data on food intake was self-reported. In the case of branded products, internet research was used to verify whether an organic variant of a product was commercially available. This could not be done in the case of loose or unpackaged goods, such as fruit and vegetables, or for takeaway foods. As a result, it is impossible to completely rule out the presence of errors in the data on organic food intake. Furthermore, the dietary assessment only covered a short time frame of four days. Although it should be possible to use data collected over a four-day period to differentiate people who eat no organic foods from those with a low or high organic food intake, individual participants may still have been wrongly categorised because the probability of eating organic food increases with the length of the assessment. Finally, self-reported data (on organic foods) may be biased by social desirability. This could have led to an overestimation of organic food intake.

Nevertheless, the EsKiMo II module is the first to provide population-based data on the proportion of organic food consumed by children aged between six and eleven in Germany. The high proportion of children who consume organic foods suggests that health, environmental and ethical motives play a role in the choice of food selected by families with children.

## Key statements

- Around 63% of 6- to 11-year-old girls and boys eat organic food.
- On average, around 8.0% of the food eaten by children who consume any amount of organic food is produced by organic farming.
- The proportion of organic food in total consumption increases with higher socioeconomic status.
- Vegetables and fruit are among the most commonly consumed organic produce in children.

## Figures and Tables

**Table 1:** Proportion of organic food in the diet by sex, age and socioeconomic status (n=584 girls, n=606 boys) Source: EsKiMo II (2015–2017)

No consumption of organic food			Organic food consumption			
			≤8.0%*		>8.0%*	
%		(95% CI)	%	(95% CI)	%	(95% CI)
**Total** **(girls and boys)**	**36.8**	**(32.8**–**40.9)**	**43.0**	**(39.0–46.9)**	**20.2**	**(16.8**–**23.6)**
**Sex** Girls Boys	35.3 38.3	(29.9–40.7) (32.4–44.1)	42.9 43.0	(37.5–48.2) (37.7–8.4)	21.8 18.7	(17.0–26.6) (14.5–22.9)
**Age group** 6–8 years 9–11 years	35.8 37.9	(30.1–41.5) (32.6–43.1)	43.0 42.9	(37.5–8.5) (37.7–8.1)	21.2 19.3	(16.6–25.7) (14.4–24.1)
**Socioeconomic status** Low Medium High	65.1 34.0 19.5	(53.7–76.4) (28.5–39.4) (14.7–24.3)	29.5 46.2 47.6	(17.8–1.3) (40.8–51.5) (40.8–54.3)	5.4 19.9 33.0	(0.1–10.7) (16.6–24.1) (26.4–39.5)

CI=confidence interval

*The 8.0% threshold is the mean level of organic food consumption found among children who eat organic produce.
